# Does sleep disturbance predicts posttraumatic stress disorder and depression among college students during COVID-19 lockdown? A longitudinal survey

**DOI:** 10.3389/fpubh.2022.986934

**Published:** 2022-09-13

**Authors:** Dongfang Wang, Jingbo Zhao, Shuyi Zhai, Haoxian Ye, Luowei Bu, Fang Fan

**Affiliations:** ^1^Guangdong Key Laboratory of Mental Health and Cognitive Science, Ministry of Education Key Laboratory of Brain Cognition and Educational Science, Centre for Studies of Psychological Applications, School of Psychology, South China Normal University, Guangzhou, China; ^2^Department of Psychology, School of Public Health, Southern Medical University, Guangzhou, China; ^3^Faculty of Medicine, McGill University, Montreal, QC, Canada

**Keywords:** sleep disturbance, depression, posttraumatic stress disorder, college students, COVID-19

## Abstract

**Aim:**

To examine the cross-sectional and longitudinal associations between self-reported sleep disturbances, posttraumatic stress disorder (PTSD) and depression in a large cohort of Chinese adolescents experiencing the COVID-19 pandemic.

**Methods:**

Participants were 67905 Chinese college students in the two-wave longitudinal web-based survey during early COVID-19 outbreak (Time1, T1: Feb 3rd to 10th, 2020) and initial remission period (Time2, T2: March 24th to April 3rd, 2020). The Youth Self Rating Insomnia Scale (YSIS), 6-Item Impact of Event Scale (IES-6), and 9-Item Patient Heath Questionnaire (PHQ-9) were used to assess adolescents' sleep, PTSD, and depressive symptoms, respectively, at T1 and T2.

**Results:**

Self-reported PTSD and depression prevalence at T1 were 34.6% and 21.6% respectively. While depressive symptoms worsened as the lockdown time increased, while PTSD symptoms decreased. After adjusting for confounding factors, sleep disturbance and sleep deprivation at T1 were significantly associated with increased PTSD and depressive symptoms at T2. Furthermore, sleep disturbance and sleep deprivation also predicted the new onset and persistence of PTSD and depression.

**Conclusion:**

Sleep disturbance predicts the development and persistence of PTSD and depression. Early assessment and treatment of sleep disturbance may be an important strategy for prevention and intervention of PTSD and depression in adolescents after experiencing the special public health emergency.

## Background

COVID-19 pandemic outbreak and subsequent quarantine have adverse impact on mental health among public (1, 2). Results from the recent meta-analysis including studies from 17 countries indicated high level of depression (28%) during the pandemic (3). Another meta-analysis including 19 studies documented that the after the COVID-19 outbreak, the prevalence of depression in general population ranged from 14.6 to 48.3%, as well as the rate of PTSD ranged from 7 to 53.8% (4). Therefore, paying greater attention to individuals' mental health during the pandemic era is urgently needed. To decrease the risk of pandemic-related stress symptoms and depression, screening for PTSD, depression and research into factors related to this psychological distress is imperative.

Past studies also have highlighted how pandemic lockdown increases various sleep disturbances, such as insomnia, poor sleep quality, and decrease sleep duration (5–7). It is well established that sleep disturbance, a modifiable behavior, is strongly associated with increased risk of mental health problems. There is limited longitudinal research however that has examined the extent to which disturbed sleep predicts the development of PTSD and depression. One study examined insomnia and daytime sleepiness at 1 month of 102 victims of motor vehicle accidents, suggesting that these sleep disturbances significantly predicted PTSD at 12 months after the trauma (8). However, another study in 453 Dutch military service members has only established the association between the presence of predeployment nightmares with an increased risk for developing PTSD symptoms at 6 months postdeployment among, while predeployment of insomnia did not (9). Moreover, a one-year prospective study of 1,573 adolescent earthquake survivors reported that sleep disturbance could predict the development and persistence of PTSD and depression after controlling for demographics and earthquake exposure (10). Based on these studies, the temporary association between sleep disturbance and subsequent PTSD and depression appears to be inconsistent, which may be explained by differences in sample and traumatic events experienced.

Meanwhile, most of previous related studies evaluated sleep disturbance as a long-term risk factor (about a year and more) for PSTD and depression. In fact, clinical risk assessments are more focused on determining whether one would be affected by a disorder in the near future. Recognizing and treating sleep disturbances is of particular importance during stress provoking time such as the COVID-19. If an individual's sleep disturbance can be measured during the early period of a pandemic, it may predict the possibility of developing PTSD and depression during home-isolation. Therefore, understanding the degree to which disrupted sleep predicts short-term risk in necessary. Moreover, most of previous related studies involved a small sample size, suggesting sampling bias that can further obscure this relationship. There is also lack of epidemiological data from large samples on relationship between the sleep disturbance, PTSD, and depression.

Furthermore, studies have suggested that compared with general population, students are more susceptible to suffer from the psychological impact of the pandemic (11). To contain the spread of the pandemic, the Chinese government has announced the closure of schools, colleges/ universities, and other educational institutions in spring 2020 during which all students were asked to stay at home and pursue their studies online. The direct impact of the confinement includes physical inactivity, lack of academic schedule, and excessive digital use, all of which increased the risk of mental health issues among adolescents (12). For college students, the uncertainty of future career or academic opportunities due to the lockdown further increased their psychological stress (13). Accordingly, paying greater attention to college students' mental health during the pandemic era is urgently needed.

Collectively, the current study attempts to investigate PTSD and depression among college students during COVID-19, as well as to enhance understanding of sleep disturbance as a risk factor for the onset and persistence of PTSD and depression in a 2-month follow-up sample. This study aims to investigate: (a) the prevalence rates of PTSD and depression in Chinese college students during the pandemic; (b) whether sleep disturbance can be cross-sectionally associated with the current and follow-up PTSD and depression; and (c) whether baseline sleep disturbance can predict the change of PTSD and depression.

## Methods

### Participants and procedure

Using the repeated cross-sectional study design, we conducted a two-wave longitudinal web-based survey on mental health in college students from 22 colleges/universities in Guangdong provinces, Southern China. Since winter 2019, COVID-19 has rapidly spread across China, with the total number of confirmed cases increased to 80,905 by March 10, 2020. During this period of the COVID-19 outbreak (Time1, T1: from February 3 to February 10, 2020), 164,101 participants completed the online questionnaires. Since March 10, the pandemic in China has been brought basically under control, with the number of newly confirmed cases showing a consistent downward trend nationwide. The second wave of the survey was conducted in the same population during the remission period of COVID-19 (Time2, T2: from March 24 to April 3, 2020).

**Figure 1:**
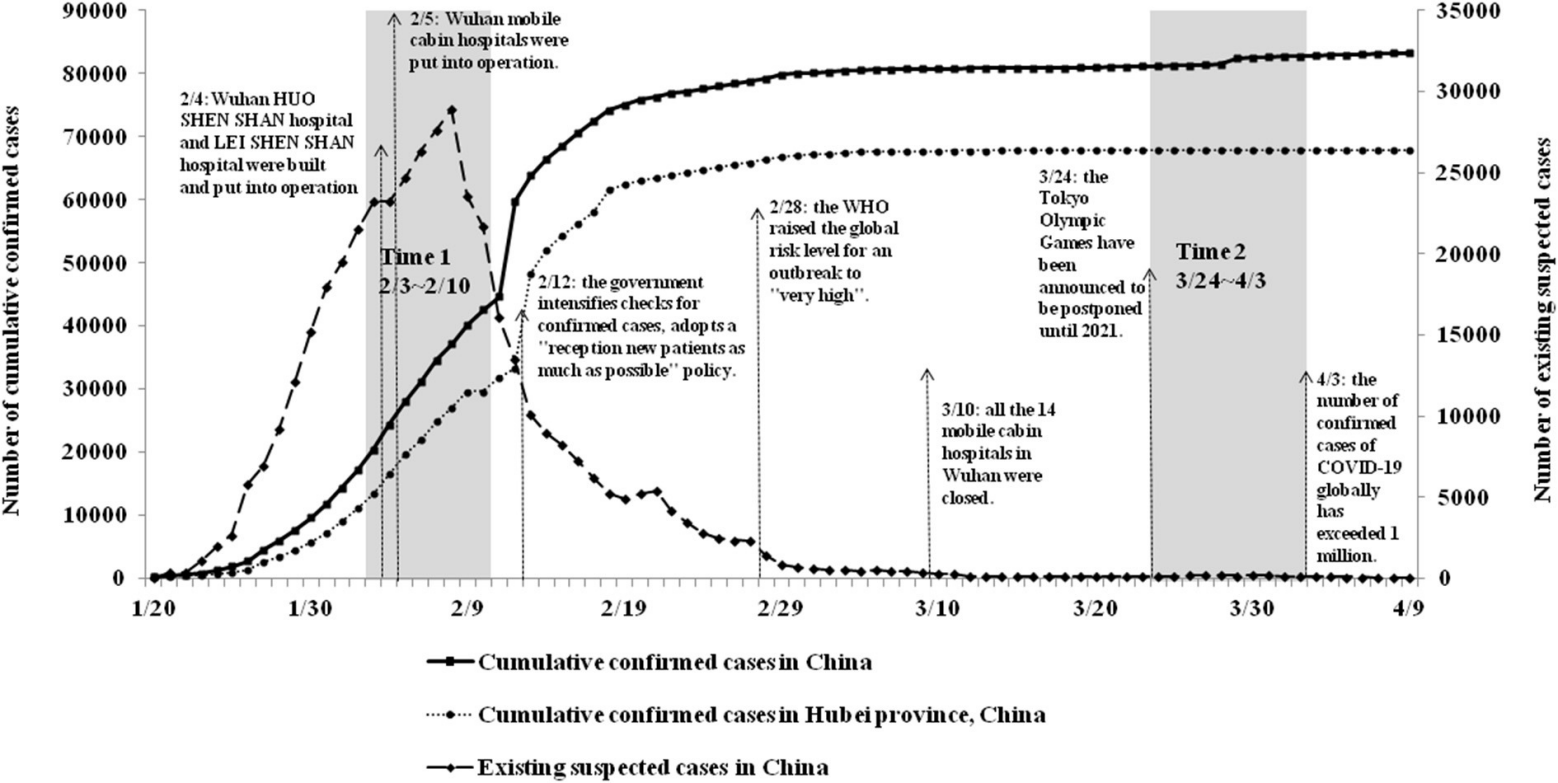
The details of study design and sample procedures.

Participants in this study were home isolation during the study period. We push questionnaires to target colleges/ universities through our self-built information website and official WeChat account, and the questionnaires are distributed to college students through psychological counseling centers of each school. Participants scan the Quick Response (QR) code on their mobile phone to complete an online survey at home. Participants needed to submit an online informed consent form before the survey and have a right to withdraw freely during test period. Through data integration, A total of 67905 college students (31.3% male) participated in all two web-based surveys and provided complete data on all measures. The details of the study design and sample procedures have also been described elsewhere (7, 14). Figure 1 shows the details of study design and sample procedures.

This study was supported by the local education bureau, and approved by the Human Research Ethics Committee of South China Normal University (SCNU-PSY-2020-01-001). We also opened a psychological hotline (the ‘Xinqing' hotline) to provide free psychological assistance services to participants if they need during lockdown.

### Measures

#### Sample characteristics

Sample characteristics included sex [1= Male; 2= Female], age, grade [1= Freshman; 2= Sophomore; 3= Junior; 4= Senior; 5= Graduate], residence location [1= Rural; 2= Urban], ethnicity [1= Han (the ethic majority in China); 2= Others], single child status [1= Yes; 2= No], history of physical illness [1= Yes; 2= No], and history of mental illness [1= Yes; 2= No].

#### COVID-19 related factors

COVID-19 related factors included the severity of the pandemic in the place of residence [1= Mild; 2= Moderate; 3= Severe], confirmed COVID-19 cases in the community or village [1= Yes; 2= No], and relatives or friends being infected with COVID-19 [1= Confirmed/ suspected; 2= No]. The severity of the pandemic in the place of residence was categorized according to the World Health Organization (WHO) guideline in early 2020 (15). Severe risk areas have more than 10,000 cumulative COVID-19 confirmed cases (e.g., Hubei province); moderate risk areas have 1,000 to 9,999 cumulative confirmed cases (e.g., Guangdong province); and the remaining provinces in China are mild risk areas with less than 1,000 cumulative confirmed cases (e.g., Sichuan province).

#### Sleep disturbance

Four items were drawn from the Chinese Version of Youth Self-Rating Insomnia Scale (YSIS) (16) to measure sleep status over the last two weeks at T1. Three items were used to assess insomnia symptoms, including difficulty initiating sleep, difficulty maintaining sleep, and early morning awakening. Participants had options to answer 1 = Never, 2 = <1 times/week, 3 = 1-2 times/week, 4 = 3-5 times/week, or 5= 5-7 times/week. Meanwhile, one item - “How would you rate your overall sleep quality over past two weeks?” was used to assess subjective sleep quality, with response options of 1= very good, 2 = good, 3 = fair, 4 = poor, 5 = very poor. In the present study, sleep disturbance was defined as having any one of the following four sleep symptoms: difficulty initiating sleep (≥3 times/week), difficulty maintaining sleep (≥3 times/week), early morning awakening (≥3 times/week), or poor/very poor sleep quality. This definition has been used in previous publications using the similar question on sleep disturbance (7, 10). The Cronbach' s alpha of four items was 0.77 at T1. Sleep duration was asked as such, “how many hours of sleep did you get at night over past two weeks?” Responses to items were recorded: 1 ≤ 5 h, 2 = 5–6 h, 3 = 6–7 h, 4 =7–8 h, or 5 ≥ 8 h. Sleep time < 6 h per night was considered as sleep deprivation (17, 18).

#### PTSD

The 6-item Impact of Event Scale (IES-6) was used to assess PTSD over past one week in the form of two surveys (19). It was anchored to the COVID-19 pandemic in this study, and clustering into three dimensions: intrusion (e.g., I thought about the COVID-19 pandemic when I didn't mean to), avoidance/numbing (e.g., I tried not to think about the COVID-19 pandemic), and hyperarousal (e.g., I had trouble concentrating because of the COVID-19 pandemic). Each item was rated on a five-point Likert scale from 0- not at all to 4- extremely. The average score of 0–1.09 indicates normal, between 1.09 and 1.5 shows stress symptoms, 1.5 or greater may be diagnosed with PTSD (20). The Chinese version of IES-6 demonstrated satisfactory reliability, as well as be widely used in the Chinese population (21, 22). The Cronbach' s alpha was 0.80 at T1 and 0.82 at T2 in this study.

#### Depression

The nine item Patient Health Questionnaire (PHQ-9) was used to measure depression over past 2 weeks at two surveys (23). Each item included four choices: 0- not at all, 1- several days, 2- more than halt the days, and 3-nearly every day. The total score ranges from a scale of 0-27, with higher total score indicating more severe symptoms of depression. Psychometric properties of the PHQ-9 have been described in the Chinese population (24). A score of 7 was identified as the optimal cut-off point for detecting clinical level of depression in Chinese population (24). The Cronbach' s alpha was 0.87 and 0.91 in two surveys, respectively.

#### Data analyses

All data analyses were conducted using IBM SPSS Statistics version 23.0. The McNemar's test was used to examine the differences in the prevalence rates of PTSD and depression between T1 and T2. In the present study, we detect four trajectories of PTSD and depression that refer to previous studies (7, 25): Persistent group (both IES-6 average score ≥1.5/ both PHQ-9 score ≥ 7); Remission group (baseline IES-6 average score ≥1.5/ PHQ-9 score ≥ 7, the second IES-6 average score <1.5/ PHQ-9 score < 7); New onset group (baseline IES-6 average score <1.5/ PHQ-9 score <7, the second IES-6 average score ≥1.5/ PHQ-9 score ≥ 7); and Resistance group (both IES-6 average score <1.5/ both PHQ-9 score < 7). A series of univariate logistic regression analyses were performed to determine the associations between each sleep variable and PTSD and depression. Further, sample characteristics and COVID-19 related factors were included to adjust for their potential confounding effects in the multivariate regression models. For all regression analyses, we excluded the one sleep-related item from PHQ-9 (Item 3: Trouble falling or staying asleep, or sleeping too much) to control the effect of collinearity. Odds ratio (OR) and 95% confidence interval (CI) were used to quantify the strength of the association.

## Results

### Description of the sample

The participants aged between 16.0 and 25.0 year-old, with the mean age of 20.23 (1.63) year-old. Among 67905 students, approximately two-third were female (68.7%, *n* = 46,635) and most were undergraduate (96.0%, *n* = 65,200). Meanwhile, 60.0% (*n* = 40,713) lived in urban areas, 98.0% (*n* = 66,517) were of Han ethnicity, 20.8 % (*n* = 14,140) were of the only child in their family. More detail characteristics have been reported elsewhere (7).

### Prevalence of sleep disturbance, PTSD and depression

**Figure 2:**
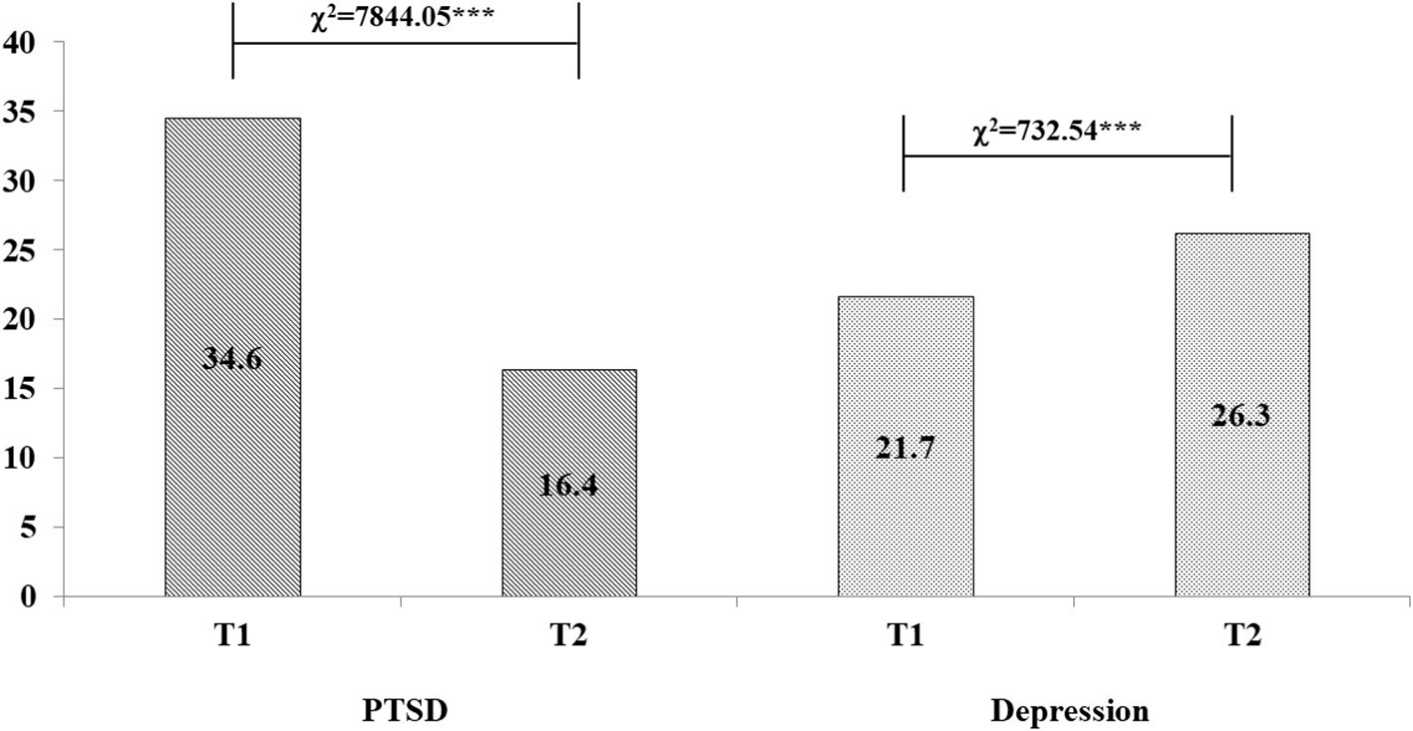
Prevalence rates of PTSD and depression at two follow-ups (%), ***p < 0.001.

The prevalence of sleep deprivation (<6 h per night) and overall sleep disturbance were 2.9 and 8.5% at T1, respectively. The prevalence of PTSD was 34.6% at T1 and has significantly decreased at T2 (16.4%, χ^2^ = 7,844.05, *p* < 0.001). The prevalence of depression at T1 was 21.7% with significantly increase at T2 (26.3%, χ^2^ = 732.54, *p* < 0.001), see Figure 2. Table 1 depicts the relationship between demographics, COVID-19 related factors, PTSD and depression among college students.

**Table 1:** Prevalence of PTSD and depression by demographics and COVID-19 related factors (*n* = 67,905).

			**PTSD** ^a^ **[%]**		**Depression** ^b^ **[%]**	
**Variables**		**Total [n (%)]**	**T1**	**T2**	**T1**	**T2**
Sex	Male	21,270 (31.3)	34.8	20.3	19.2	25.0
	Female	46,635 (68.7)	34.5	14.6	22.8	26.9
	χ^2^		0.55	352.15^***^	110.40^***^	26.22^***^
Grade	Freshman	23,921 (35.2)	32.5	15.9	20.4	24.2
	Sophomore	20,533 (30.2)	34.7	16.6	21.8	26.0
	Junior	14,540 (21.4)	36.1	16.4	22.4	27.8
	Senior	6,206 (9.1)	37.1	17.2	25.2	31.8
	Graduate	2,705 (4.0)	38.9	16.2	20.3	26.6
	χ^2^		101.90^***^	8.18	77.98^***^	167.81^***^
Residence location	Rural	27,192 (40.0)	35.6	17.3	20.9	25.7
	Urban	40,713 (60.0)	34.0	15.7	22.2	26.7
	χ^2^		18.89^***^	31.50^***^	16.01^***^	9.22^**^
Ethnicity	Han	66,517 (98.0)	34.6	16.3	21.7	26.3
	Others	1,388 (2.0)	36.5	18.4	21.7	27.7
	χ^2^		2.13	4.49^*^	0	1.49
Single child status	No	14,140 (20.8)	35.2	16.6	22.0	26.6
	Yes	53,765 (79.2)	32.5	15.3	20.3	25.1
	χ^2^		34.41^***^	13.43^***^	21.08^***^	13.23^***^
History of physical illness	Yes	335 (0.5)	37.0	20.6	26.0	31.0
	No	67,570 (99.5)	34.6	16.3	21.6	26.3
	χ^2^		0.86	4.41^*^	3.67	3.89
History of mental illness	Yes	534 (0.8)	35.8	19.3	49.4	52.6
	No	67,371 (99.2)	34.6	16.3	21.4	26.1
	χ^2^		0.32	3.37	244.50^***^	192.16^***^
The severity of the epidemic in the place of residence	Mild	6439 (9.5)	34.7	16.1	20.8	25.8
	Moderate	61030 (89.9)	34.6	16.4	21.8	26.4
	Severe	436 (0.6)	39.4	18.1	22.0	27.5
	χ2		4.59	1.39	3.34	1.17
Confirmed COVID-19 cases in the community or village	Yes	4,679 (6.9)	39.2	20.1	29.4	34.8
	No	63,226 (93.1)	34.3	16.1	21.1	25.7
	χ2		47.52^***^	52.23^***^	175.32^***^	187.41^***^
Relatives or friends being infected with	Confirmed/suspected	735 (1.1)	45.3	25.2	34.7	37.8
COVID-19	No	67,152 (98.9)	34.5	16.3	21.5	26.2
	χ^2^		38.32^***^	43.79^***^	75.71^***^	52.27^***^

* p < 0.05,

** p < 0.01,

*** p < 0.001.

a PTSD calculated using the IES-6, with a clinical cut-off score of 1.5.

b Depression calculated using the PHQ-9, with a clinical cut-off score of 7.

**Figure 3:**
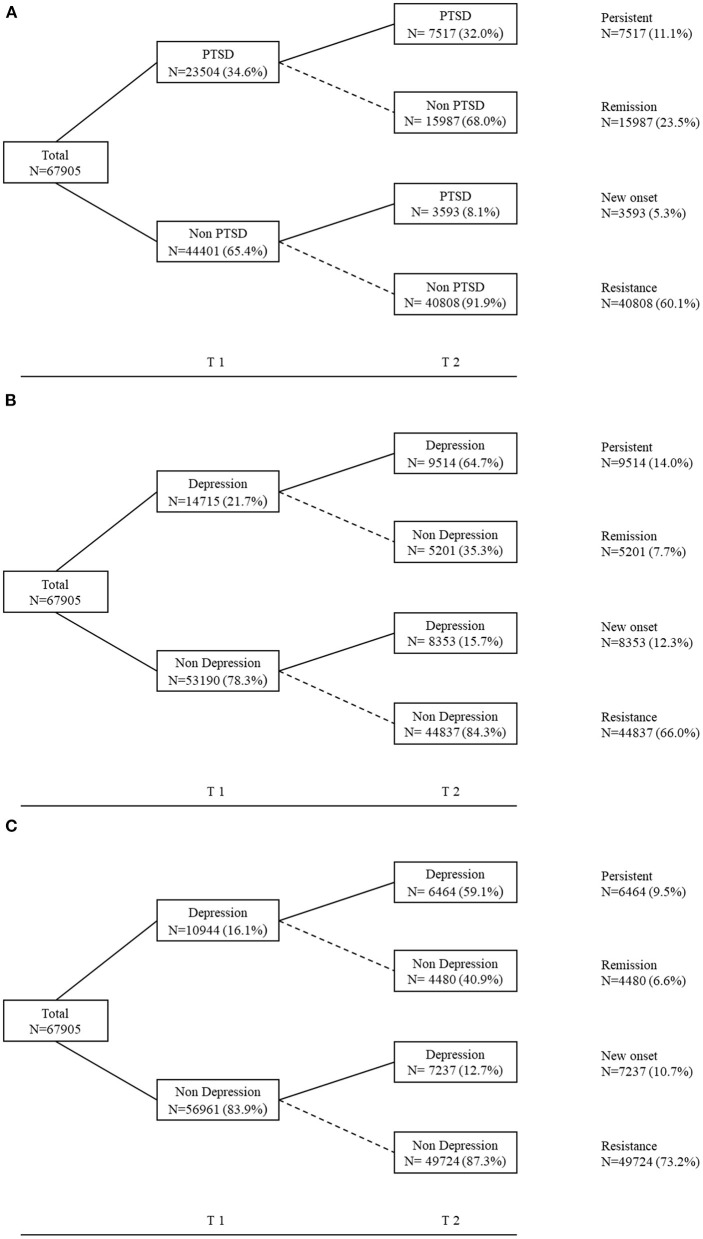
Trajectory changes of mental health problems among colleges students during the pandemic. **(A)** The trajectory of PTSD; PTSD calculated using the IES-6, with a clinical cut-off score of 1.5. **(B)** The trajectory of depression; Depression calculated using the PHQ-9, with a clinical cut-off score of 7. **(C)** The trajectory of depression; Depression calculated using the PHQ-8 (exclude the sleep-related item), with a clinical cut-off score of 7.

Figure 2 illustrates the trajectory changes of PTSD and depression among colleges students during the pandemic. As shown in Figure 3A, 11.1% of college students who had PTSD at T1 continued to have PTSD at T2, these participants are classified as the persistent group. While 5.3% of those who only developed PTSD at T2, these participants are named as the new onset group. The other two trajectories included 23.5% participants in remission group, and 60.1% in the resistance group. As shown in Figure 3B, four trajectory changes of depression as follow: persistent (14.0%), remission (7.7%), new onset (12.3%), resistance group (66.0%). Figure 3C also showed four trajectory changes of depression base on the cut-off point of 7 after excluded the sleep-related item from PHQ-9.

### Cross-sectional associations of sleep with PTSD and depression

The prevalence rates of PTSD and depression at baseline significantly increased in the presence of various sleep disturbances. College students who reported having sleep disturbance were more likely to have PTSD (52.3% vs 33.0%; OR = 2.23; 95% CI = 2.11–2.35) and depression (47.7 vs. 13.2%; OR = 6.01; 95% CI = 5.68–6.36). After adjusting for sample characteristics and COVID-19 related factors, difficulty initiating sleep (PTSD: OR = 3.05; 95% CI = 2.85–3.27; Depression: OR = 7.20; 95% CI = 6.83–7.59), difficulty maintaining sleep (PTSD: OR = 2.71; 95% CI = 2.47–2.97; Depression: OR = 5.20; 95% CI = 4.92–5.48), early morning awakening (PTSD: OR = 3.31; 95% CI = 2.93–3.74; Depression: OR = 6.50; 95% CI = 6.11–6.92)≥ 3 nights per week, poor sleep quality (PTSD: OR = 2.81; 95% CI = 2.56–3.09; Depression: OR = 12.10; 95% CI = 10.97–13.35), and overall sleep disturbance (PTSD: OR = 2.25; 95% CI = 2.13–2.37; Depression: OR = 5.78; 95% CI = 5.46–6.12) were all still significantly associated with increased risk of PTSD and depression. Sleep deprivation (PTSD: OR = 1.71; 95% CI = 1.56–1.87; Depression: OR = 3.55; 95% CI = 3.22–3.92) was also significantly associated with PTSD and depression (see Table 2).

**Table 2:** Prevalence of PTSD and depression at T1 and their associations with sleep disturbance at T1.

		**PTSD at T1**			**Depression at T1** ^#^	
**Sleep at T1**	**%**	**Crude OR (95% CI)**	**Adjust OR (95% CI) ^a^**		**%**	**Crude OR (95% CI)**	**Adjust OR (95% CI) ^a^**
**Sleep duration**						
>8 h	32.8	1.00	1.00	14.6	1.00	1.00
7–8 h	34.8	1.09 (1.05,1.13)^***^	1.09 (1.05,1.13)^***^	14.6	1.01 (0.96,1.06)	1.02 (0.98,1.07)
6–7 h	39.9	1.36 (1.29,1.44)^***^	1.36 (1.29,1.43)^***^	23.4	1.80 (1.69,1.92)^***^	1.82 (1.70,1.94)^***^
<6 h	45.7	1.73 (1.57,1.90)^***^	1.71 (1.56,1.87)^***^	38.2	3.63 (3.29,4.00)^***^	3.55 (3.22,3.92)^***^
**Difficulty initiating sleep**						
Never/ <1 night/week	27.1	1.00	1.00	7.5	1.00	1.00
1–2 nights/week	42.8	2.01 (1.94,2.08)^***^	2.02 (1.95,2.09)^***^	18.5	2.81 (2.66,2.97)^***^	2.79 (2.64,2.94)^***^
≥3 nights/week	52.6	2.98 (2.79,3.19)^***^	3.05 (2.85,3.27)^***^	37.2	7.36 (6.99,7.75)^***^	7.20 (6.83,7.59)^***^
**Difficulty maintaining sleep**						
Never/ <1 night/week	29.9	1.00	1.00	10.4	1.00	1.00
1–2 nights/week	44.2	1.86 (1.80,1.92)^***^	1.85 (1.79,1.91)^***^	22.6	2.52 (2.39,2.65)^***^	2.51 (2.38,2.64)^***^
≥3 nights/week	53.6	2.82 (2.48,2.98)^***^	2.71 (2.47,2.97)^***^	38.3	5.36 (5.08,5.65)^***^	5.20 (4.92,5.48)^***^
**Early morning awakening**						
Never/ <1 night/week	30.6	1.00	1.00	11.2	1.00	1.00
1–2 nights/week	48.9	2.17 (2.09,2.26)^***^	2.15 (2.07,2.23)^***^	28.6	3.19 (3.03,3.36)^***^	3.18 (3.02,3.35)^***^
≥3 nights/week	59.5	3.34 (2.96,3.77)^***^	3.31 (2.93,3.74)^***^	45.6	6.67 (6.27,7.10)^***^	6.50 (6.11,6.92)^***^
**Subjective sleep quality**						
Very good/ Good	30.5	1.00	1.00	10.1	1.00	1.00
Normal	47.4	2.06 (1.98,2.14)^***^	2.07 (1.99,2.15)^***^	33.2	4.44 (4.23,4.64)^***^	4.38 (4.18,4.58)^***^
Poor/ Very poor	54.8	2.76 (2.51,3.03)^***^	2.81 (2.56,3.09)^***^	58.6	12.63 (11.46,13.92)^***^	12.10 (10.97,13.35)^***^
**Sleep disturbance** ^b^						
No	33.0	1.00	1.00	13.2	1.00	1.00
Yes	52.3	2.23 (2.11,2.35)^***^	2.25 (2.13,2.37)^***^	47.7	6.01 (5.68,6.36)^***^	5.78 (5.46,6.12)^***^

*** p < 0.001.

a Adjusting for age, sex, grade, residence location, ethnicity, only single child, history of physical/ mental illness, and COVID-19 related factors.

b Sleep disturbance = difficulty initiating sleep (≥3 nights/week), difficulty maintaining sleep (≥3 nights/week), Early morning awakening (≥3 nights/ week), or poor/very poor sleep quality.

# Depression calculated using the PHQ-8 (exclude the sleep-related item), with a clinical cut-off score of 7.

### Longitudinal associations of sleep with PTSD and depression

As shown in Table 3, the unadjusted odds of PTSD and depression at T2 have significantly increased with difficulty initiating sleep, difficulty maintaining sleep, early morning awakening, poor sleep quality, and with reduced sleep duration. After controlling for sample characteristics, COVID-19 related factors, and baseline PTSD or depression, difficulty initiating sleep (PTSD: OR = 1.82; 95% CI = 1.67–1.98; Depression: OR = 2.64; 95% CI = 2.51–2.78), difficulty maintaining sleep (PTSD: OR = 1.64; 95% CI = 1.46–1.83; Depression: OR = 2.08; 95% CI = 1.97–2.21), early morning awakening (PTSD: OR = 1.82; 95% CI = 1.58–2.09; Depression: OR = 2.22; 95% CI = 2.07–2.38)≥3 nights per week, poor sleep quality (PTSD: OR = 1.78; 95% CI = 1.59–1.99; Depression: OR = 2.72; 95% CI = 2.43–3.03), and overall sleep disturbance (PTSD: OR = 1.50; 95% CI = 1.39–1.60; Depression: OR = 2.16; 95% CI = 2.02–2.30) at T1 also remained significantly associated with increased odds for PTSD and depression at T2. Sleep deprivation (PTSD: OR = 1.69; 95% CI = 1.51–1.90; Depression: OR = 1.93; 95% CI = 1.73–2.16) was also a significant predictor for PTSD and depression.

**Table 3:** Prevalence of PTSD and depression at T2 and their associations with sleep disturbance at T1.

		**PTSD at T2**			**Depression at T2** ^#^	
**Sleep at T1**	**%**	**Crude OR (95% CI)**	**Adjust OR (95% CI) ^a^**	**%**	**Crude OR (95% CI)**	**Adjust OR (95% CI) ^b^**
**Sleep duration**						
>8 h	15.1	1.00	1.00	18.7	1.00	1.00
7–8 h	16.3	1.10 (1.05,1.15)^***^	1.04 (0.99,1.09)	18.8	1.00 (0.96,1.04)	0.99 (0.95,1.04)
6–7 h	20.1	1.42 (1.33,1.52)^***^	1.25 (1.16,1.34)^***^	27.0	1.60 (1.51,1.70)^***^	1.33 (1.24,1.42)^***^
<6 h	26.8	2.06 (1.86,2.29)^***^	1.69 (1.51,1.90)^***^	40.9	3.00 (2.73,3.30)^***^	1.93 (1.73,2.16)^***^
**Difficulty initiating sleep**						
Never/ <1 night/week	12.8	1.00	1.00	12.1	1.00	1.00
1–2 nights/week	20.0	1.71 (1.64,1.79)^***^	1.42 (1.35,1.48)^***^	23.2	2.19 (2.08,2.29)^***^	1.72 (1.63,1.81)^***^
≥3 nights/week	26.7	2.48 (2.30,2.69)^***^	1.82 (1.67,1.98)^***^	38.9	4.61 (4.41,4.83)^***^	2.64 (2.51,2.78)^***^
**Difficulty maintaining sleep**						
Never/ <1 night/week	13.8	1.00	1.00	15.1	1.00	1.00
1–2 nights/week	21.6	1.72 (1.64,1.79)^***^	1.46 (1.39,1.53)^***^	26.6	2.05 (1.95,21.4)^***^	1.59 (1.51,1.67)^***^
≥3 nights/week	26.0	2.19 (1.97,2.43)^***^	1.64 (1.46,1.83)^***^	38.9	3.59 (3.41,3.78)^***^	2.08 (1.97,2.21)^***^
**Early morning awakening**						
Never/ <1 night/week	14.0	1.00	1.00	15.9	1.00	1.00
1–2 nights/week	24.6	2.00 (1.91,2.10)^***^	1.59 (1.51,1.67)^***^	31.7	2.45 (2.33,2.57)^***^	1.73 (1.64,1.83)^***^
≥3 nights/week	30.4	2.68 (2.36,3.06)^***^	1.82 (1.58,2.09)^***^	44.5	4.24 (4.00,4.51)^***^	2.22 (2.07,2.38)^***^
**Subjective sleep quality**						
Very good/ Good	13.9	1.00	1.00	14.9	1.00	1.00
Normal	23.9	1.94 (1.85,2.03)^***^	1.55 (1.48,1.63)^***^	35.7	3.17 (3.03,3.30)^***^	2.05 (1.95,2.14)^***^
Poor/ Very poor	28.4	2.45 (2.21,2.72)^***^	1.78 (1.59,1.99)^***^	53.4	6.55 (5.96,7.20)^***^	2.72 (2.43,3.03)^***^
**Sleep disturbance**						
No	15.5	1.00	1.00	17.7	1.00	1.00
Yes	25.9	1.91 (1.80,2.04)^***^	1.50 (1.39,1.60)^***^	46.6	4.06 (3.84,4.30)^***^	2.16 (2.02,2.30)^***^

*** p < 0.001.

a Adjusting for age, sex, grade, residence location, ethnicity, only single child, history of physical/ mental illness, COVID-19 related factors, and PTSD at T1.

b Adjusting for age, sex, grade, residence location, ethnicity, only single child, history of physical/ mental illness, COVID-19 related factors, and depression at T.

# Depression calculated using the PHQ-8 (exclude the sleep-related item), with a clinical cut-off score of 7.

### Sleep disturbance predicting change of PTSD and depression

As shown in Table 4, sleep duration and sleep disturbances were used to predict changes in PLEs. sample characteristics and COVID-19 related factors were adjusted for their potential confounding effects. Compared with resistance group, adjusted OR of sleep disturbance was 1.71 (95% CI = 1.52–1.93) for new onset PTSD, as well as 2.68 (95% CI = 2.46–2.91) for new onset depression. Meanwhile, compared to the remission group, adjusted OR of overall sleep disturbance was 1.40 (95% CI = 1.30–1.52) for persistent PTSD, as well as 1.69 (95% CI = 1.54–1.85) for persistent depression. Sleep deprivation was also a significant predictor for new onset (PTSD: OR = 1.99; 95% CI = 1.67–2.38; Depression: OR = 2.24; 95% CI = 1.95–2.58) and persistent (PTSD: OR = 1.53; 95% CI = 1.33–1.77; Depression: OR = 1.57; 95% CI = 1.33–1.85) PTSD or depression.

**Table 4:** Sleep disturbance predicting change of PTSD or depression during COVID-19.

	**PTSD**					**Depression** ^#^			
**Sleep at T1**	**New-onset v. resistance**		**Persistent v. remission**		**New-onset v. resistance**		**Persistent v. remission**	
	**Crude OR**	**Adjust OR**	**Crude OR**	**Adjust OR**	**Crude OR**	**Adjust OR**	**Crude OR**	**Adjust OR**
	**(95% CI)**	**(95% CI) ^a^**	**(95% CI)**	**(95% CI) ^a^**	**(95% CI)**	**(95% CI) ^a^**	**(95% CI)**	**(95% CI) ^a^**
**Sleep duration**								
>8 hours	1.00	1.00	1.00	1.00	1.00	1.00	1.00	1.00
7-8 hours	1.04 (0.97,1.12)	1.01 (0.93,1.09)	1.09 (1.02,1.15)^**^	1.06 (1.00,1.13)	1.00 (0.95,1.06)	1.00 (0.94,1.05)	0.99 (0.91,1.08)	0.99 (0.91,1.08)
6-7 hours	1.36 (1.21,1.52)^***^	1.30 (1.16,1.46)^***^	1.25 (1.14,1.37)^***^	1.22 (1.11,1.33)^***^	1.34 (1.23,1.45)^***^	1.33 (1.22,1.45)^***^	1.32 (1.17,1.48)^***^	1.31 (1.17,1.48)^***^
<6 hours	2.12 (1.77,2.53)^***^	1.99 (1.67,2.38)^***^	1.59 (1.38,1.83)^***^	1.53 (1.33,1.77)^***^	2.28 (1.99,2.62)^***^	2.24 (1.95,2.58)^***^	1.60 (1.36,1.89)^***^	1.57 (1.33,1.85)^***^
**Difficulty initiating sleep**								
Never/ <1 night/week	1.00	1.00	1.00	1.00	1.00	1.00	1.00	1.00
1–2 nights/week	1.30 (1.20,1.41)^***^	1.98 (1.85,2.06)^***^	1.21 (1.13,1.30)^***^	1.31 (1.20,1.44)^***^	1.90 (1.79,2.01)^***^	1.90 (1.79,2.02)^***^	1.22 (1.10,1.35)^***^	1.22 (1.10,1.35)^***^
≥3 nights/week	1.72 (1.57,1.88)^***^	3.15 (2.96,3.35)^***^	1.50 (1.40,1.59)^***^	1.95 (1.79,2.12)^***^	3.02 (2.83,3.21)^***^	3.03 (2.84,3.22)^***^	1.86 (1.69,2.04)^***^	1.86 (1.69,2.04)^***^
**Difficulty maintaining sleep**								
Never/ <1 night/week	1.00	1.00	1.00	1.00	1.00	1.00	1.00	1.00
1–2 nights/week	1.37 (1.26,1.49)^***^	1.79 (1.69,1.90)^***^	1.29 (1.21,1.38)^***^	1.34 (1.24,1.46)^***^	1.72 (1.62,1.83)^***^	1.72 (1.62,1.83)^***^	1.28 (1.17,1.41)^***^	1.28 (1.16,1.40)^***^
≥3 nights/week	1.60 (1.44,1.78)^***^	2.52 (2.34,2.72)^***^	1.53 (1.42,1.64)^***^	1.73 (1.59,1.88)^***^	2.46 (2.28,2.64)^***^	2.44 (2.27,2.62)^***^	1.61 (1.46,1.76)^***^	1.58 (1.44,1.74)^***^
**Early morning awakening**								
Never/ <1 night/week	1.00	1.00	1.00	1.00	1.00	1.00	1.00	1.00
1–2 nights/week	1.61 (1.47,1.77)^***^	1.66 (1.51,1.83)^***^	1.41 (1.31,1.51)^***^	1.43 (1.34,1.54)^***^	2.01 (1.88,2.15)^***^	2.01 (1.88,2.15)^***^	1.27 (1.16,1.40)^***^	1.26 (1.15,1.39)^***^
≥3 nights/week	1.90 (1.67,2.17)^***^	1.92 (1.69,2.19)^***^	1.70 (1.56,1.84)^***^	1.72 (1.58,1.87)^***^	2.87 (2.62,3.14)^***^	2.83 (2.58,3.10)^***^	1.61 (1.45,1.78)^***^	1.58 (1.43,1.75)^***^
**Subjective sleep quality**								
Very good/ Good	1.00	1.00	1.00	1.00	1.00	1.00	1.00	1.00
Normal	1.65 (1.52,1.79)^***^	1.66 (1.53,1.81)^***^	1.47 (1.39,1.56)^***^	2.83 (2.68,3.00)^***^	2.37 (2.41,2.51)^***^	2.37 (2.24,2.51)^***^	1.51 (1.39,1.63)^***^	1.50 (1.39,1.63)^***^
Poor/ Very poor	2.00 (1.63,2.44)^***^	1.99 (1.62,2.44)^***^	1.67 (1.47,1.90)^***^	4.25 (3.74,4.83)^***^	3.67 (3.13,4.29)^***^	3.59 (3.07,4.21)^***^	2.02 (1.76,2.33)^***^	2.00 (1.73,2.30)^***^
**Sleep disturbance**								
No	1.00	1.00	1.00	1.00	1.00	1.00	1.00	1.00
Yes	1.68 (1.49,1.89)^***^	1.71 (1.52,1.93)^***^	1.38 (1.27,1.49)	1.40 (1.30,1.52)^***^	2.71 (2.50,2.95)^***^	2.68 (2.46,2.91)^***^	1.70 (1.56,1.87)^***^	1.69 (1.54,1.85)^***^

** p < 0.01,

*** p < 0.001.

a Adjusting for age, sex, grade, residence location, ethnicity, only single child, history of physical/ mental illness, and COVID-19 related factors.

# Depression calculated using the PHQ-8 (exclude the sleep-related item), with a clinical cutoff score of 7.

## Discussion

This study is the first large-scale longitudinal study on the relationship between sleep disturbances, PTSD and depression among college students during the COVID-19 pandemic in China. Our findings showed that the prevalence of PTSD and depression was 34.6 and 21.6% during early COVID-19 outbreak, respectively. As the lockdown progresses in China, depressive symptoms seemed to increase but PTSD symptoms seemed to decrease. Meanwhile, sleep disturbance and sleep deprivation were cross-sectional and longitudinally associated with increased risk of PTSD and depression. In addition, our data also revealed four trajectories for PTSD and depression, namely resistance, persistent, new onset, and remission, among which sleep disturbances were significant predictors of distinct PTSD and depression trajectories.

In this study, we found that 8.5% of college students had experienced sleep disturbance during pandemic outbreak. Results of previous studies varied due to the differences in sampling, measures and cut-off points. For instance, Wang and colleagues investigated 3,092 Chinese college students' sleep status using the Self-Rating Scale of Sleep (SRSS) were reported that 5.3% participants had sleep problems during the outbreak of COVID-19 (5). Studies by Zhou et al., sleep was analyzed using the Pittsburgh Sleep Quality Index (PSQI), indicated a prevalence of clinically insomnia being 23.2% during pandemic (6). During the outbreak of pandemic, 2.9% of college students have reported sleep deprivation, which is consistent with previous studies with 2.7% of college students sleeping <6 h per night during outbreak of COVID-19 (26).

Meanwhile, our data showed that about 34.6% of participants had possible clinically significant PTSD during the COVID-19 pandemic outbreak period. The prevalence is similar to the results of previous studies with a larger sample. Previous studies with same measurement scales and similar sampling time found that about 32.7% of 304,167 college students had PTSD (21). We also found that 21.6% college students had clinical level depression, which was also similar to previous studies using the same measurement scale by Ma et al. (21.1%) (27). However, the rate of PTSD has significantly decreased between the two-wave assessment periods. The result was also in line with previous studies (28). This decrease in PTSD symptoms could potentially be explained by China's effective control of the COVID-19. As time elapsed, knowledge of reliable information on COVID-19, effective prevention and control measures, enhanced medical support and a public health service systems implemented by the Chinese government through trial and error, might have contributed in reassuring people and alleviating the initial worry and fear caused by the emergence of the pandemic. However, similar to prior research (14, 29–31), depression increased from 21.7% at T1 to 26.3% at T2. Specifically, depressive symptoms have worsened as the lockdown time increased, which be explained by the chronic stress associated with unexpected changes in living patterns for college students (i.e., lacking in exercise, confinement for social distancing, and delay in returning to school) due to long-term lockdown (12), which led to increase the risk of depression.

The PTSD and depression trajectories showed that the majority of college students in the study (60.1% and 66.0 for resistance) exhibited no symptoms throughout the 2-month period post-pandemic. Meanwhile, a small percentage of the college students exhibited the trajectories of persistence, remission and new onset. This result showed that the overall PTSD and depressive symptoms is relatively low and very stable. We speculated that college students exposed to the COVID-19 pandemic exhibited acute stress responses only immediately after the public health emergency while maintained a stable trajectory of euthymia and healthy functioning. However, the presence or new onset of PTSD and depression might be related to secondary stressors during pandemic lockdown, such as lifestyle and economic disruptions (32). Having more time spent with family and reduced academic stress could contribute to symptoms remission (25).

Adjusting for socio-demographics variables and COVID-19 related factors, sleep disturbances have been cross-sectionally associated with PTSD and depression during the pandemic. Much literature has also identified that individual with sleep disturbances is at a higher risk of PTSD and depression than those without sleep complaints (33, 34). Meanwhile, different from the long-term risks identified in previous studies, this study also confirmed the strong associations between sleep disturbances and PTSD or depression within a shorter time-frame after the exposure to a special public health emergency. Specifically, these findings were expected and in line with several studies (8, 10, 35) that prospectively demonstrated the association of previous sleep disturbances with later onset and persistence of PTSD and depression. On the contrary, good sleep quality is a protective factor for PTSD and depression remission. Previous studies suggested that sleep disturbance following a trauma might amplify or prolong typical stress responses and increase the likelihood of PTSD development (36). Recent evidence has also suggested that college students with sleep disturbance are prone to maladaptive emotion regulation, resulting in the development of depressive symptoms during COVID-19 lockdown (37).

Moreover, our data also demonstrated significant cross-sectional and longitudinal associations between sleep deprivation, PTSD and depression. These results are consistent with findings prior to the pandemic, although most studies have been limited by cross-sectional design. For example, one cross-sectional study on American veterans demonstrated the significant association between sleep deprivation and increased odds of concurrent PTSD (38). Few prospective studies have shown that sleep deprivation at baseline predicted depression at 1 year later among adolescents, after adjusting for baseline depression (17). Longitudinal analysis further demonstrated that sleep deprivation could independently predict the new onset or persistence of PTSD and depression, after controlling for socio-demographics variables and COVID-19 related factors. However, the 12-month follow up by Fan et al. demonstrated that sleep deprivation could not independently predict the onset of PTSD and depression (10). The inconsistency of these results may be due to the difference in follow up time. The two surveys in this study were separated by only 2 months, and the effect of sleep deprivation was relatively stable. Another explanation could be that insufficient sleep among college students during pandemic lockdown could be related to their excessive media use at night, which could be their mean to acquire more COVID-19 related information. Indeed, exposure to media coverage of the COVID-19 was found may be a risk factor for individual mental health during outbreaks (27).

Finally, this study makes a unique contribution to the literature by examining the association between sleep disturbances, PTSD and depression in a college student sample during the COVID-19 pandemic. Our findings suggest that recognizing and treating sleep disturbance is especially vital during stress-inducing periods such as the pandemic. For groups in isolation at home, we can appropriately increase sleep duration and improve sleep quality through measures such as exercise training (39) or cognitive behavioral therapy for insomnia (CBT-i) (40). However, several limitations also should be noted. In this study, sleep, PTSD, and depression were all assessed by self-reported questionnaires rather than face-to-face clinical interviews, which may have led to reporting bias caused by participants various psychiatric states and recollection inaccuracy. In the form of web-based questionnaires, it is also difficult to answer participants' questions in a timely manner during the survey process, which might reduce the reliability of the data. Meanwhile, there is a significant sex imbalance in our data, which may bias the current results. Finally, some confounding factors such as negative life events were not considered in our study, which may also impact the current results.

## Conclusion

This is the first study to survey both cross-sectional and longitudinal associations of sleep disturbance with PTSD and depression in a large cohort of college students exposed to COVDI-19. Our data showed that sleep disturbance was cross-sectional and prospectively associated with an increased risk of PTSD and depression. It also predicted changes of PTSD and depression. Furthermore, the current study shed light on the notion that assessing several simple symptoms related to sleep problems may be a quick and effective way to screen individuals at increased risk of PTSD and depression. Therefore, assessment and treatment of sleep disturbance as early as possible may be an important strategy for prevention and intervention of mental disorders in individuals after exposure to a public health emergency.

## Data availability statement

Publicly available datasets were analyzed in this study. The raw data supporting the conclusions of this article will be made available by the authors.

## Ethics statement

The studies involving human participants were reviewed and approved by the Ethics Board of the South China Normal University. The patients/participants provided their written informed consent to participate in this study.

## Author contributions

DW, JZ, and FF: conceptualization. DW: methodology, formal analysis, and writing—original draft. DW, JZ, HY, and LB: data curation. JZ, SZ, and FF: writing–review and editing. All authors contributed to the article and approved the submitted version.

## Funding

The present study was funded by the National Natural Science Foundation of China (Grant No. 31871129), Research on the Processes and Repair of Psychological Trauma in Youth, Project of Key Institute of Humanities and Social Sciences, MOE (Grant No. 16JJD190001), and Guangdong Province Universities and Colleges Pearl River Scholar Funded Scheme (GDUPS 2016).

## Conflict of interest

The authors declare that the research was conducted in the absence of any commercial or financial relationships that could be construed as a potential conflict of interest.

## Publisher's note

All claims expressed in this article are solely those of the authors and do not necessarily represent those of their affiliated organizations, or those of the publisher, the editors and the reviewers. Any product that may be evaluated in this article, or claim that may be made by its manufacturer, is not guaranteed or endorsed by the publisher.
